# Exploring Manual Interventions for Infantile Colic: A Scoping Review of the Evidence

**DOI:** 10.3390/children12091246

**Published:** 2025-09-17

**Authors:** Roberto Tedeschi, Federica Giorgi

**Affiliations:** 1 Independent Researcher, 40100 Bologna, Italy; 2 Physical Medicine and Rehabilitation Unit, University Hospital Policlinico Sant’Orsola-Malpighi, 40121 Bologna, Italy; federica.giorgi15@gmail.com

**Keywords:** infantile colic, manual therapy, massage, craniosacral therapy

## Abstract

**Background:** Infantile colic affects up to 40% of otherwise healthy infants and can severely distress caregivers. Manual therapies are increasingly employed as non-pharmacological options, yet their effectiveness and safety remain uncertain. **Methods:** A scoping review was conducted in accordance with Joanna Briggs Institute methodology and reported following PRISMA-ScR. Five databases (MEDLINE, CENTRAL, Scopus, PEDro, Web of Science) were searched from December 2024 to May 2025 without restrictions at the search stage; however, only English-language randomised controlled trials published from 2012 onwards were included at the eligibility stage to ensure consistency and focus on the most recent body of evidence. Randomised controlled trials (RCTs) evaluating hands-on interventions for infants ≤ 6 months with colic were eligible. Two reviewers independently screened records, charted data, and grouped outcomes narratively. **Results:** Seven RCTs investigated abdominal massage, paediatric Tuina, craniosacral therapy, chiropractic manipulation, osteopathic light touch, reflexology, and acupressure. Five trials reported statistically or clinically significant reductions in daily crying (0.6–6.6 h) compared with usual care or sham. Three studies also documented meaningful gains in sleep duration (1.1–2.8 h). Parent-reported satisfaction improved in most interventions. No serious adverse events were recorded, although safety monitoring was inconsistently reported. Substantial heterogeneity in diagnostic criteria, outcome measures, and intervention dose precluded meta-analysis. **Conclusions:** Low-force manual therapies may offer modest short-term relief for colicky infants and improve parental experience, with an apparently favourable safety profile. However, methodological variability and small sample sizes limit certainty. Standardised protocols, objective outcome measures, and robust adverse-event surveillance are priorities for future research.

## 1. Introduction

Infantile colic is one of the commonest functional disorders in early life, typically emerging between the second and twelfth week after birth with paroxysms of inconsolable crying in an otherwise healthy infant. It was classically defined by Wessel’s “rule of threes”—crying for ≥3 h day^−1^, on ≥3 days week^−1^, for ≥3 consecutive weeks [1]. The more recent Rome IV criteria broaden this description to “recurrent and prolonged periods of crying, fussing or irritability” in infants <5 months, thereby acknowledging that distress does not always conform to rigid temporal cut-offs [2]. Contemporary epidemiological syntheses report global point prevalences of 10–20% in the first six months of life [3], with crying duration peaking at six weeks before declining sharply after the third month [4]. Cross-national comparisons involving nearly 8700 infants indicate striking cultural variation, from only 6.7% of Danish infants meeting colic criteria to 28% in the United Kingdom [5]. Although colic is self-limiting, its impact on families and healthcare systems is substantial. Excessive crying is associated with heightened caregiver frustration, sleep deprivation, and relationship strain [6], and robust evidence links maternal depression and anxiety both concurrently and prospectively with persistent infant crying [7]. In primary care, colic accounts for up to one in six consultations during the first quarter of life and frequently triggers formula changes, pharmacological prescriptions, and even hospital referrals, thereby inflating healthcare costs [8]. The aetiology of colic remains elusive. Multifactorial models implicate gastrointestinal immaturity, dysbiosis of the gut microbiota, altered neurodevelopmental regulation and psychosocial factors, yet none provides a definitive explanation [9]. This uncertainty is mirrored by a heterogeneous therapeutic landscape. Dietary approaches—such as maternal exclusion of cow’s milk protein or the use of hydrolysed formulas—show inconsistent benefit and are hampered by small, methodologically limited trials [10]. Probiotic supplementation has been examined both prophylactically and therapeutically, but recent Cochrane reviews conclude that evidence is insufficient to justify routine use [11,12]. Pharmacological agents fare no better: systematic reviews find no convincing efficacy for simethicone or other pain-relieving drugs and highlight potential risks [13]. Complementary options such as acupuncture are likewise characterised by small, heterogeneous studies that preclude firm conclusions [14]. In this context, manual therapies—including chiropractic and osteopathic manipulation, craniosacral therapy, massage, and reflexology—have attracted growing interest. These techniques share the premise that gentle, hands-on interventions can modulate neuromuscular and autonomic function, thereby reducing crying episodes. A 2012 Cochrane review reported modest reductions in daily crying but judged the underlying evidence to be of low quality owing to high risk of bias and marked heterogeneity [15]. Since then, the evidence base has expanded: a 2022 umbrella review catalogued an increasing number of randomised controlled trials (RCTs) but reiterated concerns regarding blinding and small sample sizes [16]. Notable recent RCTs include a Danish trial of chiropractic care (*n* = 194), which reported a mean reduction of 30 min day^−1^ compared with control [17]; a multicentre European trial of light-touch osteopathy (*n* = 66) that found no superiority over sham touch [18]; and a Spanish study of craniosacral therapy (*n* = 58) showing almost three hours’ shorter crying duration by day 24 relative to parental advice alone [19]. Collectively, these trials underscore both the promise of manual care and the persistent uncertainty surrounding its effectiveness.

A critical evidence gap therefore persists. The last comprehensive synthesis predates many of the RCTs cited above, and existing narrative updates have neither applied contemporary risk-of-bias tools nor investigated how factors such as intervention dose, practitioner background, or diagnostic criteria influence outcomes. Consequently, clinicians and parents remain uncertain about the clinical value and safety of manual therapies, while researchers lack clear priorities for future work. This scoping review aims to map and synthesise the existing evidence on manual therapies for infantile colic published since 2012. Given the growing interest in hands-on interventions such as chiropractic care, osteopathy, craniosacral therapy, massage, and reflexology, our objective is to provide a comprehensive overview of the types of interventions studied, their reported outcomes, and the methodological characteristics of the trials conducted to date. By identifying key trends, knowledge gaps, and sources of heterogeneity across the literature, we intend to offer a solid foundation for future systematic reviews and to support clinicians and caregivers in understanding the current research landscape on manual therapies for infantile colic.

## 2. Methods

This scoping review was designed in accordance with the methodological framework proposed by the Joanna Briggs Institute (JBI) [20] for the conduct of scoping reviews. To enhance transparency and ensure methodological rigour, we followed the recommendations outlined in the PRISMA-ScR (Preferred Reporting Items for Systematic Reviews and Meta-Analyses Extension for Scoping Reviews) statement [21].

### 2.1. Review Question

We formulated the following research question: “What is the scope and nature of the current evidence on manual therapy interventions for the management of infantile colic?”

### 2.2. Eligibility Criteria

Studies were eligible for inclusion if they met the following Population, Concept, and Context (PCC) criteria.

**Population (P):** Infants aged 0–6 months formally diagnosed with infantile colic, according to either Wessel’s criteria (“rule of threes”) or the Rome IV diagnostic criteria. Studies were included regardless of gender, gestational age, or feeding method (breast-fed/formula-fed). Only studies involving otherwise healthy infants were considered.

**Concept (C):** The review considered studies investigating any form of manual therapy aimed at reducing the frequency, intensity, or duration of colic symptoms. Eligible interventions included, but were not limited to:

• Chiropractic techniques
• Osteopathy and craniosacral therapy
• Paediatric massage and Tuina
• Reflexology
• Acupressure or touch-based therapeutic interventions

The interventions could be administered by healthcare professionals or trained caregivers and delivered either in clinical settings or at home.

**Context (C):** Any healthcare or home-based setting was eligible, including outpatient paediatric clinics, osteopathic/chiropractic practices, hospitals, or family homes. Studies from any country and healthcare system were included, provided that the full text was available in English.

### 2.3. Exclusion Criteria

The following studies were excluded:

Studies involving infants with underlying neurological, gastrointestinal, or structural anomalies other than colic, or where the diagnosis of colic did not meet accepted clinical criteria (Wessel or Rome IV).

Studies that investigated non-manual treatments (e.g., pharmacological, dietary, or orthotic interventions) without a manual therapy component.

Studies that did not include primary outcomes related to colic symptoms, such as crying duration, frequency of episodes, parental perception of symptom improvement, or sleep duration.

Non-randomised studies, observational cohorts, case reports, qualitative studies, protocols, editorials, letters to the editor, and systematic or narrative reviews.

Studies not published in English or without an available full text were excluded to ensure consistent analysis.

### 2.4. Search Strategy

A structured and database-specific search was carried out across five major biomedical platforms to identify relevant studies on manual therapies for infantile colic. The search strategies were tailored to each database’s indexing system and search functionalities, combining both controlled vocabulary (e.g., MeSH terms) and free-text keywords where applicable. No filters were applied regarding publication year, country, or healthcare setting, in order to maximise the comprehensiveness of the evidence retrieved.

MEDLINE (PubMed):

((“Infant, Newborn” [MeSH] OR newborn* OR infant* OR baby OR babies) AND (“Colic” [MeSH] OR colic* OR “infantile colic” OR “baby colic” OR “excessive crying”) AND (“Manipulation, Chiropractic” [MeSH] OR “Musculoskeletal Manipulations” [MeSH] OR “Manual Therapy” [MeSH] OR chiropractic OR osteopath* OR “craniosacral therapy” OR massage OR reflexology OR acupressure OR “manual intervention*” OR “physical manipulation” OR “manual therap*” OR “touch therapy”))

Cochrane Central Register of Controlled Trials (CENTRAL):

(newborn* OR infant* OR baby OR babies) AND (colic OR “infantile colic” OR “baby colic” OR “excessive crying”) AND (chiropractic OR osteopath* OR “craniosacral therapy” OR massage OR reflexology OR acupressure OR “manual therapy” OR “manual intervention*” OR “physical manipulation” OR “touch therapy”)

Scopus:

TITLE-ABS-KEY ((newborn* OR infant* OR baby OR babies) AND (colic OR “infantile colic” OR “baby colic” OR “excessive crying”) AND (chiropractic OR osteopath* OR “craniosacral therapy” OR massage OR reflexology OR acupressure OR “manual therapy” OR “manual intervention*” OR “physical manipulation” OR “touch therapy”))

PEDro:

This was queried using a simplified strategy due to its restricted search syntax. The term “*infantile colic*” was used as the primary keyword in the title and abstract fields. The following filters were applied to refine the results:

• Subdiscipline: *Paediatrics*
• **Method:** Randomised Controlled Trial (RCT)
• **Therapy type:** Manual therapy, Massage, or Manipulative therapy

Given the limited functionality of Boolean operators and controlled vocabulary in PEDro, manual screening of titles and abstracts was subsequently performed to identify studies evaluating manual interventions such as chiropractic care, osteopathy, craniosacral therapy, or reflexology. Only trials involving infants aged 0–6 months with a diagnosis of colic were considered eligible.

Web of Science:

TS = ((newborn* OR infant* OR baby OR babies) AND (colic OR “infantile colic” OR “baby colic” OR “excessive crying”) AND (chiropractic OR osteopath* OR “craniosacral therapy” OR massage OR reflexology OR acupressure OR “manual therapy” OR “manual intervention*” OR “physical manipulation” OR “touch therapy”))

### 2.5. Study Selection

All references retrieved between December 2024 and May 2025 were exported to Zotero, where automatic and manual checks were used to remove duplicates. Study selection then proceeded in two successive stages: (i) a title-and-abstract screen and (ii) a full-text appraisal of citations deemed potentially eligible. Each stage was completed independently by two reviewers; any discordant decisions were discussed and, when necessary, adjudicated by a third reviewer. Throughout, the workflow was aligned with the PRISMA 2020 guidance for transparent reporting and with current JBI recommendations for scoping reviews.

### 2.6. Data Extraction and Data Synthesis

Key data—including study design, characteristics of the participants, intervention details, outcome measures, and main findings—were extracted using a standardised data charting form developed a priori to ensure uniformity across studies. Outcomes were organised into thematic categories to allow for cross-study comparisons. A descriptive synthesis was used to identify common patterns and to highlight knowledge gaps in the existing literature. When relevant, quantitative results were reported to illustrate overarching trends. This structured approach enabled a comprehensive and coherent interpretation of the available evidence in accordance with the aims of the review.

## 3. Results

**Figure 1:**
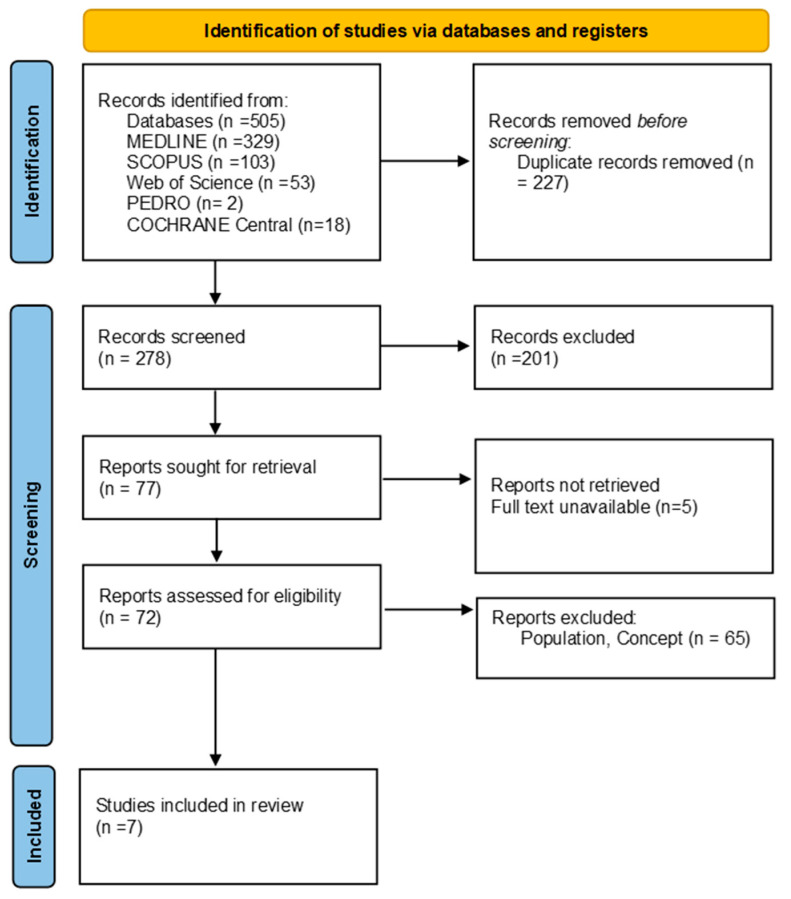
Preferred reporting items for systematic reviews and meta-analyses 2020 (PRISMA) flow diagram.

As illustrated in the PRISMA 2020 flow diagram (Figure 1), the initial database search yielded a total of 505 records. Following the screening and eligibility assessment, 498 studies were excluded based on the predetermined criteria, and 7 studies met the inclusion criteria and were retained for analysis (see Table 1).

### 3.1. Crying Duration

All seven included trials assessed changes in the duration of daily crying, making this the most frequently evaluated outcome across the body of evidence. Most studies demonstrated a reduction in crying time following manual therapy interventions, although the magnitude of improvement and statistical robustness varied. In Moghaddam et al.’s [22] three-arm trial, infants receiving Hugo-point acupressure showed the most marked decrease in daily crying, with a mean reduction of 6.62 h compared to 3.55 h in the abdominal massage group and 3.92 h in the control group. These differences were statistically significant and suggest a potential dose–response relationship favouring more targeted interventions.

Castejón-Castejón et al. [19] reported similarly encouraging findings following craniosacral therapy, with crying hours reduced by nearly three hours per day by the end of the third week. These results were accompanied by improvements in both sleep and colic severity scores. In contrast, Carnes et al. [18] found no meaningful difference in crying time between osteopathy and a well-matched sham comparator, with a negligible adjusted difference of 2.2 min per day. Holm et al. [17] reported a modest but borderline non-significant between-group reduction of 0.6 h in favour of chiropractic manipulation after two weeks of treatment, with *p* = 0.066 after adjustment.

Zhao et al. [24] observed a substantial drop in daily crying episodes and total crying time following paediatric Tuina therapy, while Karataş et al. documented a 41% reduction in crying duration after just one session of foot reflexology, compared to a 15% reduction in the placebo group. Sheidaei et al. [25] also reported a significant decline of 62 min in daily crying following parental-delivered abdominal massage, suggesting feasibility and efficacy even with minimal intervention. Overall, although methodological heterogeneity was present, the evidence supports a general trend towards reduced crying duration following hands-on interventions.

### 3.2. Crying Frequency

Only three studies explicitly assessed the frequency of crying episodes as a discrete outcome. Among these, Zhao et al. [24] provided the most rigorous data, with infants in the Tuina group reducing their daily crying episodes from a baseline mean of 5.1 to 1.9 by the end of the five-day intervention period. This change was both statistically significant and clinically meaningful. Although Sheidaei et al. did not quantify crying frequency directly, the reduction in total crying time and parent-reported diaries suggested a parallel decrease in how often crying episodes occurred. Karataş et al. [23] captured this outcome indirectly through caregiver assessments of irritability and behavioural settling, noting greater improvements in the reflexology group compared to placebo. While fewer studies examined this variable, preliminary evidence suggests that manual therapies may not only shorten the duration of crying but also reduce its frequency, contributing to a more stable behavioural rhythm in affected infants.

### 3.3. Sleep Duration

Sleep duration was assessed in four of the included studies, often in conjunction with reductions in crying. Improvements in sleep were generally aligned with reductions in colic severity, suggesting a bidirectional relationship between pain relief and sleep regulation. The most substantial gains were reported by Castejón-Castejón et al., who found an increase of 2.8 h in total sleep duration per day following craniosacral therapy, a change that achieved statistical significance and was highly valued by caregivers.

In Sheidaei et al.’s trial, sleep duration increased by an average of 1.1 h per day in the massage group, compared to 0.3 h in the control group. Although Moghaddam et al. did not provide precise numerical values, the authors reported significant improvements in sleep quality and duration in both the massage and acupressure groups. Conversely, Holm et al. did not observe significant changes in sleep patterns between the chiropractic and sham groups, possibly reflecting the limited intensity or duration of the intervention. Overall, manual therapies appear to offer modest but meaningful improvements in sleep for infants with colic, particularly when delivered in a repetitive and parent-centred fashion.

### 3.4. Parent-Reported Perception of Improvement

Several studies incorporated parent-reported outcomes, including perceived improvement, satisfaction, and confidence in managing colic. These subjective measures provide insight into caregiver experience and expectations, which are especially relevant in paediatric interventions. In Holm et al.’s [17] trial, 35% of parents in the chiropractic group reported a clinically meaningful improvement in their infant’s symptoms, compared to 20% in the sham group, despite minimal differences in objective crying time.

Carnes et al. [18] used the Karitane Parent Confidence Scale to evaluate caregiver reassurance, finding similar improvements in both intervention and sham groups. This suggests a strong non-specific or contextual effect, potentially linked to therapist–parent interaction or the act of focused attention on the infant. Zhao et al. [24] documented greater parental satisfaction in the Tuina group, which mirrored the larger reductions in crying frequency and duration. Castejón-Castejón et al. [19] also found high levels of reported improvement in caregiver perception, consistent with the objective changes observed in colic severity and sleep quality. These findings underscore the importance of including subjective outcomes in paediatric trials, as parental perception often guides treatment-seeking behaviour and adherence.

### 3.5. Safety and Adverse Events

Three trials explicitly addressed safety outcomes, with no reports of serious adverse events or clinically significant distress related to the interventions. Karataş et al. [23], Holm et al. [17], and Carnes et al. [18] all confirmed the absence of complications during or after treatment sessions, even in very young infants. These findings reinforce the general safety and tolerability of manual therapies in this population, particularly when administered by trained professionals using low-force or gentle techniques. Nonetheless, the absence of standardised adverse event reporting protocols in most studies limits the strength of this conclusion. Future trials should incorporate systematic monitoring of both short- and long-term safety outcomes to provide a more comprehensive risk–benefit profile (Table 2).

**Table 2:** Summary of Outcomes Across Included RCTs Evaluating Manual Therapies for Infantile Colic.

Study (Author, Year)	Crying Duration	Crying Frequency	Sleep Duration	Parental Perception	Safety Reported
Moghaddam et al., 2022 [22]	↑	NR	↑	NR	NR
Holm et al., 2021 [17]	→	NR	→	↑	✓
Castejón-Castejón et al., 2022 [19]	↑	NR	↑	↑	NR
Sheidaei et al., 2016 [25]	↑	→	↑	↑	NR
Zhao et al., 2023 [24]	↑	↑	NR	↑	NR
Karataş et al., 2021 [23]	↑	→	NR	↑	✓
Carnes et al., 2024 [18]	→	NR	→	→	✓

**Legend:** ↑: Outcome achieved (statistically and/or clinically significant improvement). →: No significant improvement. NR: Not reported. ✓: Safety assessed and no adverse events reported.

## 4. Discussion

The present scoping review maps the trajectory of research on manual therapies for infantile colic and highlights both encouraging signals and persistent methodological uncertainty. Across the seven randomised controlled trials included, five reported statistically or clinically meaningful reductions in daily crying time when some forms of hands-on intervention—most commonly abdominal massage, paediatric Tuina, craniosacral techniques, or acupressure—were compared with usual care. These findings mirror the direction, though not the strength, of the 2012 Cochrane review that first suggested a potential benefit of manual treatment, yet rated the underlying evidence as low quality. This aligns with findings from Dobson et al. (2012) [15], who noted a trend toward improvement but highlighted small sample sizes and inconsistent blinding. More recently, a meta-analysis by Park et al. (2023) [16] confirmed modest effects yet underscored high heterogeneity and risk of bias.

What distinguishes the contemporary evidence base is its broadened therapeutic palette and a modest diversification of geographical settings, with trials now originating from Europe, the Middle East, and East Asia [24]. Nevertheless, the field still lacks a convergent signal robust enough to establish clinical effectiveness beyond a reasonable doubt.

A closer look at individual trials underscores the heterogeneity that continues to impede definitive conclusions. Moghaddam [22] and colleagues’ sizeable reductions in crying duration after Hugo-point acupressure and abdominal massage contrast sharply with the null findings of Carnes et al. [18], who tested a rigorously blinded, light-touch osteopathic protocol against an equally tactile sham. Likewise, Holm et al.’s [17] chiropractic study demonstrated an unadjusted benefit that eroded once baseline crying, infant age, and clinic site were controlled, suggesting that baseline characteristics can strongly influence apparent treatment effects. This reinforces earlier observations by Miller and Newell (2012) [26], who stressed the need for stratification by age and baseline symptom severity. The largest absolute improvement—a near three-hour reduction in crying accompanied by a 2.8 h gain in sleep—was observed after craniosacral therapy delivered over three weeks, but the sample comprised fewer than sixty infants and lacked a sham comparator. Such variability in both intervention content and trial design complicates attempts to synthesise outcomes quantitatively and points to an urgent need for standardised treatment protocols and core outcome sets. As also highlighted by Tanrıverdi et al. (2023) [27], the lack of a core outcome set for colic impedes cumulative knowledge generation.

Sleep emerged as the outcome most frequently improving in tandem with reduced crying, reinforcing the clinical intuition that better regulation of arousal and circadian rhythms accompanies symptomatic relief in colic. Yet the measurement of sleep relied almost exclusively on caregiver diaries rather than objective monitoring such as actigraphy, leaving open the possibility of expectancy effects [19]. Parent-reported satisfaction, assessed in five trials, tended to be higher than would be predicted by changes in crying alone, an observation that speaks to the multifaceted nature of parental distress and to the therapeutic value of empathetic practitioner–parent interactions. Parental perception may therefore constitute both an outcome of interest and a potential confounder, particularly in trials where blinding is partial or absent [17].

Safety reporting was generally reassuring, with three trials explicitly stating that no adverse events occurred. However, none employed a structured adverse-event monitoring framework, and follow-up durations rarely exceeded four weeks. Given sporadic case reports linking high-velocity spinal manipulation in neonates to adverse neurological events, rigorous prospective safety surveillance remains essential, especially for techniques involving the cervical spine [28].

From a mechanistic standpoint, several plausible pathways might explain the modest benefits seen with low-force manual techniques. Gentle cutaneous stimulation has been shown to activate C-tactile afferents, facilitating vagal modulation and dampening sympathetic arousal; these effects align with the observation of improved sleep and reduced irritability. Field (2019) [29] demonstrated that moderate-pressure infant massage enhances vagal tone, which may partly explain such improvements. In addition, abdominal massage and Tuina may aid gastrointestinal motility or gas clearance, thereby addressing one of the hypothesised gut-related triggers of colic. Yet these mechanistic propositions remain speculative; none of the trials incorporated physiological markers such as heart-rate variability, gastric transit time, or stool microbiota composition [30]. Embedding such measures in future studies could help disentangle specific effects from contextual factors.

Several constraints should temper the interpretation of these findings. First, scoping reviews are designed to map the breadth of evidence rather than to generate pooled estimates of effect; consequently, no meta-analysis was undertaken. Second, the review limited inclusion to English-language RCTs published from 2012 onward, potentially omitting earlier or non-English work and thereby introducing language and time-lag bias. Third, heterogeneity in diagnostic criteria (Wessel versus Rome IV) and outcome definitions limited comparability across studies. Fourth, most trials employed caregiver diaries to record crying, a method highly susceptible to recall bias and inter-individual variability. Fifth, although two reviewers independently screened and charted data, the search was confined to five bibliographic databases and did not systematically explore grey literature or trial registries, so unpublished negative studies may have remained undetected. Finally, risk-of-bias assessments were beyond the descriptive remit of this scoping exercise; however, previous systematic reviews consistently cite inadequate blinding and small sample sizes as recurrent concerns, suggesting that the certainty of evidence mapped here is likely low to moderate at best.

### Clinical Practice Implications

The findings of this scoping review offer cautious yet valuable insights for clinical practice in the management of infantile colic. While the overall quality of evidence remains limited, several studies report meaningful improvements in crying duration, sleep patterns, and parental satisfaction following the application of manual therapies such as abdominal massage, craniosacral therapy, chiropractic care, paediatric Tuina, and reflexology. These interventions appear to be well tolerated, with no serious adverse events reported in the short term when delivered by appropriately trained professionals using gentle techniques.

Clinicians—including physiotherapists, osteopaths, chiropractors, and paediatric manual therapists—may consider integrating such approaches into their treatment repertoire, particularly when conventional strategies (e.g., dietary changes, parental reassurance) prove insufficient. However, it is imperative that practitioners clearly communicate to caregivers the limitations of current evidence, the variability in outcomes, and the largely supportive—rather than curative—nature of these treatments.

Importantly, the therapeutic alliance between clinician and parent, along with the provision of focused attention to the infant in a calm setting, may itself contribute to the observed benefits. As such, manual therapy should not be viewed in isolation but rather as part of a multimodal, family-centred strategy. Incorporating parental education and self-management strategies, such as teaching abdominal massage techniques, may enhance both short- and long-term outcomes.

Until more robust and standardised evidence becomes available, clinicians should individualise care, monitor outcomes carefully, and prioritise interventions that combine safety, feasibility, and caregiver engagement. Ultimately, the goal remains to reduce infant distress while empowering families with non-pharmacological options that promote comfort, bonding, and confidence.

## 5. Conclusions

This scoping review highlights the growing but still methodologically fragile body of evidence surrounding manual therapies for infantile colic. Across seven randomised controlled trials conducted over the past decade, various hands-on approaches—including abdominal massage, craniosacral therapy, reflexology, paediatric Tuina, and chiropractic techniques—have shown promising but inconsistent results in reducing crying duration, improving sleep, and enhancing parental satisfaction. Notably, while many studies report improvements, the variability in treatment protocols, outcome definitions, and methodological quality limits the strength and generalisability of conclusions.

Safety findings are overall reassuring, with no serious adverse events reported; however, the absence of structured adverse event monitoring across most trials warrants caution. Moreover, the influence of contextual factors, such as parental expectations and caregiver–practitioner interaction, may partially explain some of the perceived benefits and should not be overlooked in both clinical and research settings.

Manual therapies may offer modest, short-term relief for some infants with colic, especially when integrated into a broader, family-centred care model. Nonetheless, current evidence is insufficient to support routine use without careful case-by-case consideration. Future research should aim to improve standardisation, incorporate physiological markers and long-term outcomes, and rigorously assess both efficacy and safety. Only through such high-quality investigation can the true therapeutic potential of manual interventions in infantile colic be fully understood and responsibly applied in practice.

## Figures and Tables

**Table 1:** Summary of RCTs Evaluating the Effectiveness of Manual Therapies for Infantile Colic.

Study (First Author, Year, Design)	Methods *(Setting, Participants, Intervention/Comparator)*	Key Numerical Results	Outcomes Assessed
**Moghaddam 2022—three-arm RCT** [22]	*Iran; home-based.*	Mean change in daily crying (baseline → 4 wk):	Crying duration (h day^−1^); sleep hours.
	135 breast-fed infants, 1–3 mo.	• Acupressure—6.62 ± 4.84 h	
	① Abdominal massage by mothers (15 min each evening × 4 wk).	• Massage—3.55 ± 3.12 h	
	② Hugo-point acupressure (2 min, 3 × day × 4 wk).	• Control—3.92 ± 4.02 h	
	③ Routine advice only.	***p*****< 0.05** for group effect.	
		Sleep duration increased significantly in both active groups.	
**Holm 2021—single-blind RCT** [17]	*Denmark; chiropractic clinic.*	Crying reduction at 2 wk: 1.5 h (treatment) vs. 1.0 h (sham).	Crying duration; “clinically relevant” response (≥1 h drop); sleep hours; parental satisfaction.
	194 infants, 2–14 wk (96 int/98 sham).	Unadjusted Δ—0.6 h (95% CI—1.1 to—0.1; *p* = 0.026) but not significant after covariate adjustment (*p* = 0.066).	
	Chiropractic assessment ± individualised light-force manipulation (5 min, twice-weekly for 2 wk) vs. sham handling.		
**Carnes 2024—multicentre RCT** [18]	*UK/CH/AUS osteopathy practices.*	Mean crying day^−1^ at day 14: 124 ± 69 min (osteopathy) vs. 115 ± 49 min (sham).	Crying minutes; Karitane Parent Confidence Scale; parent-reported improvement.
	66 infants < 10 wk (32 osteopathy/34 sham).	Adjusted Δ + 2.2 min (95% CI—20 to 25; *p* = 0.849).	
	Light-touch osteopathic techniques (10–20 min, 1–4 sessions) vs. purposive sham touch of equal dose.		
**Castejón-Castejón 2022—RCT** [19]	*Spain; outpatient paediatric rehab.*	At day 24:	Crying hours; sleep hours; Infant Colic Severity Questionnaire.
	58 infants < 90 d (29 CST/29 advice).	• Crying ↓ 2.94 h day^−1^ (95% CI 2.30–3.58; *p* < 0.001).	
	Craniosacral therapy (30–40 min, 1–3 sessions over 3 wk) vs. detailed caregiver advice on positioning, burping, etc.	• Sleep ↑ 2.80 h day^−1^ (95% CI 1.73–3.85; *p* < 0.001).	
		• Colic-severity score improved by 17.24 pts (*p* < 0.001).	
**Karataş 2021—placebo-controlled RCT** [23]	***Turkey**; university clinic.*	Crying duration 24 h post-treatment fell by 41% (median 85 → 50 min) vs. 15% in placebo (88 → 75 min); ***p*** **< 0.01**.	Crying minutes; parent-reported fussiness; sleep onset latency.
	45 infants, 1–3 mo (20 reflexology/25 placebo touch).	No adverse events.	
	Foot reflexology: 3–5 min relaxation + 12–15 min stimulation of digestive and CNS reflex points; single session.		
**Zhao 2023—RCT** [24]	*China; hospital outpatient.*	Cry episodes day^−1^: 5.1 ± 1.0 → 1.9 ± 1.1 (Tuina) vs. 5.0 ± 1.2 → 3.9 ± 1.3 (control); ***p*** **< 0.001**.	Crying frequency; total crying minutes; caregiver satisfaction.
	90 infants, 1–3 mo (45 Tuina/45 routine care).	Crying time mirrored episode data.	
	Pediatric Tuina massage (once daily, 5 d).		
**Sheidaei 2016—RCT** [25]	*Iran; primary-care setting.*	Mean crying day^−1^ dropped 62 min (massage) vs. 27 min (control); ***p*** **< 0.01**.	Crying minutes; crying episodes; quiet-sleep hours.
	60 infants, 1–3 mo (30 parent-delivered abdominal massage/30 routine care).	Sleep increased 1.1 h vs. 0.3 h.	
	Massage: 15 min, once daily × 2 wk.		

Legend: *CNS* = central nervous system; *CST* = craniosacral therapy; *d* = days; *h* = hours; *min* = minutes; *mo* = months; *N* = sample size; *RCT* = randomised controlled trial; *Tuina* = traditional Chinese paediatric massage; *wk* = weeks.

## Data Availability

The original contributions presented in the study are included in the article, further inquiries can be directed to the corresponding author.
